# Greater Reductions in Hepatitis B Core-Related Antigen Associated with Switching from Entecavir to Tenofovir Alafenamide Compared with Continued Entecavir Therapy: A Retrospective Observational Study

**DOI:** 10.3390/jcm15114021

**Published:** 2026-05-22

**Authors:** Masanori Fukushima, Satoshi Miuma, Yasuhiko Nakao, Ryu Sasaki, Masafumi Haraguchi, Hisamitsu Miyaaki

**Affiliations:** Department of Gastroenterology and Hepatology, Graduate School of Biomedical Sciences, Nagasaki University, Nagasaki 852-8501, Japan; miuma1002@gmail.com (S.M.); yasu.nakao@nagasaki-u.ac.jp (Y.N.); r.sasaki@nagasaki-u.ac.jp (R.S.); mharaguchi@nagasaki-u.ac.jp (M.H.); miyaaki-hi@nagasaki-u.ac.jp (H.M.)

**Keywords:** entecavir, hepatitis B core-related antigen, switching, tenofovir alafenamide

## Abstract

**Background/Objectives**: Hepatitis B core-related antigen (HBcrAg) is a surrogate marker that reflects the transcriptional activity of covalently closed circular DNA (cccDNA). However, the impact of switching nucleos(t)ide analogs on HBcrAg levels remains unclear. The current study evaluated changes in HBcrAg levels following a switch from entecavir (ETV) to tenofovir alafenamide (TAF) compared with continued ETV therapy. **Methods**: This retrospective study included patients with chronic hepatitis B who either switched from ETV to TAF between 2017 and 2022 (ETV–TAF group) or continued ETV therapy during the same period (ETV group). HBcrAg levels were measured annually, and longitudinal changes over 3 years were analyzed based on an index year defined for each patient. Propensity score matching for age, sex, HBcrAg levels, liver transplantation status, and ETV treatment duration yielded 10 patients per group. **Results**: Baseline characteristics were well balanced after matching. HBV DNA and HBsAg levels remained suppressed in both groups throughout follow-up. The ETV-TAF group showed greater declines in HBcrAg than the ETV group at year 2 (−0.20 vs. −0.10 log U/mL, *p* = 0.007) and year 3 (−0.30 vs. −0.10 log U/mL, *p* = 0.006). No virological breakthroughs occurred. **Conclusions**: Switching from ETV to TAF was associated with greater reductions in HBcrAg levels over 3 years than continued ETV therapy, even in patients with suppressed HBV DNA. These findings suggest that switching to TAF may be associated with further suppression of viral transcriptional activity reflected by HBcrAg reduction and support its potential clinical utility for achieving deeper viral suppression.

## 1. Introduction

Hepatitis B virus (HBV) infection remains a global health concern due to its progression to liver cirrhosis and hepatocellular carcinoma (HCC). Persistent viral replication and hepatic inflammation are strongly associated with hepatocarcinogenesis, and HBV infection is the leading cause of HCC worldwide [1].

The introduction of nucleos(t)ide analogs (NAs), such as entecavir (ETV) and tenofovir, has enabled sustained viral suppression and markedly improved the prognosis of patients with chronic HBV infection. Multiple studies and meta-analyses have demonstrated that NA therapy significantly reduces the risk of HCC [2]. Accordingly, reducing serum HBV DNA and hepatitis B surface antigen (HBsAg) levels is essential for minimizing HCC risk in patients with HBV infection [3].

In Japan, ETV, a guanosine analog, and tenofovir disoproxil fumarate (TDF) or tenofovir alafenamide fumarate (TAF), both adenine nucleotide analogs, are widely used as first-line therapies for HBV infection because of their potent antiviral effects and low rates of resistance [4]. However, TDF has been associated with renal tubular injury following intestinal conversion to tenofovir, which may lead to renal dysfunction, hypophosphatemia, and osteoporosis. Conversely, TAF is a prodrug with high plasma stability that is efficiently converted to tenofovir within hepatocytes and is associated with fewer adverse events [5,6]. Therefore, ETV and TAF are commonly used in clinical practice.

In addition to serum HBV DNA and HBsAg levels, several factors—including male sex, older age, low platelet count, and cirrhosis—have been identified as independent risk factors for hepatocarcinogenesis during NA therapy [7]. More recently, elevated levels of hepatitis B core-related antigen (HBcrAg) have been reported as a risk factor for HCC, even in patients with undetectable HBV DNA under NA therapy [8,9,10,11].

HBcrAg has gained attention as a surrogate marker reflecting intrahepatic covalently closed circular DNA (cccDNA) activity and remains detectable even when HBV DNA is suppressed. It is also useful for the early detection of HBV reactivation [12].

Several studies have reported that switching from ETV to TAF results in greater reductions in HBsAg levels than continued ETV therapy [13,14]. Furthermore, TDF has been associated with a lower risk of HCC than ETV [15], and TAF, as a prodrug of tenofovir, may offer similar benefits in suppressing hepatocarcinogenesis.

Despite the established association between HBcrAg and hepatocarcinogenesis, data on the effects of NA therapy on HBcrAg remain limited. Therefore, the present study aimed to evaluate changes in HBcrAg levels in patients with chronic HBV infection who continued ETV therapy and those who switched from ETV to TAF.

## 2. Materials and Methods

### 2.1. Patients and Study Design

This retrospective, observational study included patients with chronic hepatitis B who either switched from ETV to TAF between 2017 and 2022 (ETV-TAF switch group) or continued ETV during the same period (ETV continuation group).

HBcrAg levels were measured annually, and longitudinal changes were analyzed based on the index year defined for each patient. In the ETV-TAF switch group, the index year was defined as the year of switching to TAF. In the ETV group, the year 2019, corresponding to the median switching year in the ETV–TAF group, was designated as the index year to align the treatment periods between groups and enable longitudinal comparison of HBcrAg changes.

ETV was administered at 0.5 mg once daily on an empty stomach, and TAF at 25 mg once daily after meals. Patients in the ETV continuation group were required to receive ETV for at least 6 years before and after the index year. In the ETV-TAF switch group, patients were required to have received both ETV and TAF for at least 3 years each. Based on these criteria, 19 and 15 patients were included in the ETV continuation group and ETV-TAF switch group, respectively. All clinical, laboratory, and treatment adherence data were retrospectively extracted from the electronic medical records.

We used commercially available kits to test blood samples for HBsAg and hepatitis B envelope antigen (Abbott Japan, Tokyo, Japan). HBV DNA concentrations were monitored using a PCR assay (COBAS AmpliPrep-COBAS TaqMan HBV Test; Roche Diagnostics, Tokyo, Japan). HBcrAg levels were measured using a high-sensitivity chemiluminescent enzyme immunoassay (iTACT-HBcrAg assay; Fujirebio Inc., Tokyo, Japan) and were expressed as log U/mL, with a lower limit of detection of 2.1 log U/mL [12].

### 2.2. Propensity Score Matching

To adjust for baseline differences, propensity scores were calculated using age, sex, HBcrAg level at the index year, duration of ETV treatment up to the index year, and liver transplantation status. One-to-one nearest-neighbor matching without replacement was performed, yielding 10 patients per group. Balance after matching was assessed using standardized mean differences.

Because liver transplantation status was strongly associated with HBsAg levels, with all transplanted patients showing HBsAg levels < 0.05 IU/mL, both variables were not simultaneously included in the propensity score model to avoid overadjustment and collinearity. Given the greater baseline imbalance in liver transplantation status between the groups, transplantation status was prioritized over HBsAg levels in the final matching model.

### 2.3. Switching Criteria to TAF

Switching from ETV to TAF was performed in accordance with established guidelines [16]. All patients had achieved a favorable virological response to ETV prior to switching, and the decision to switch was based on the potential for future drug resistance. No patients switched due to inadequate treatment response.

### 2.4. Statistical Analysis

Continuous variables are presented as medians (interquartile ranges [IQR]), and categorical variables as counts (percentages). Between-group comparisons were performed using the Mann–Whitney U test for continuous variables and the chi-square or Fisher’s exact test for categorical variables, as appropriate.

Changes in HBcrAg from the index year were calculated at each time point and compared between groups.

Propensity scores were estimated using logistic regression with age, sex, HBsAg level, HBcrAg level, and duration of ETV treatment as covariates. One-to-one nearest-neighbor matching without replacement was applied, and post-matching balance was assessed using standardized mean differences.

A two-sided *p*-value < 0.05 was considered statistically significant. All analyses were performed using StatFlex version 7 (Artech Co., Ltd., Osaka, Japan).

## 3. Results

The baseline characteristics of 34 patients (19 in the ETV continuation group and 15 in the ETV-TAF switch group) before propensity score matching are summarized in Table 1. In the overall cohort, liver transplant recipients were observed more frequently in the ETV-TAF switch group than in the ETV continuation group. In addition, trends toward differences in sex and duration of ETV treatment up to the index year were observed between the groups. Therefore, propensity score matching was performed using age, sex, HBcrAg level at the index year, duration of ETV treatment up to the index year, and liver transplantation status.

After propensity score matching, 10 patients were assigned to each group. The median age was 68 years in the ETV continuation group and 65 years in the ETV-TAF switch group. All patients were men. The median HBcrAg level was 3.2 log U/mL in the ETV continuation group vs. 3.0 log U/mL in the ETV-TAF group. The median duration of prior ETV treatment until the index date was 75 months in the ETV continuation group vs. 82 months in the ETV-TAF group (Table 2). There were no significant differences between groups in terms of the liver functional reserve, platelet count, renal function, or FIB-4 index.

Four patients had undergone liver transplantation in the ETV group, while five patients had undergone liver transplantation in the ETV–TAF group. All transplant recipients had undergone transplantation for HBsAg-positive status and had received NA therapy before transplantation, followed by hepatitis B immunoglobulin (HBIG) for a defined period post-transplantation. No patients received HBIG after the index year (2019) or after switching to TAF. All patients received tacrolimus as immunosuppressive therapy.

**Figure 1:**
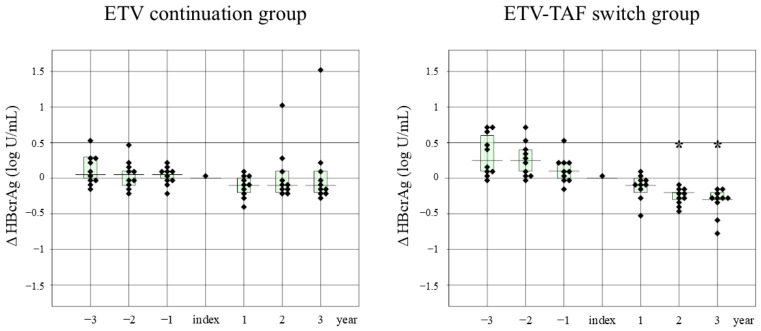
Longitudinal changes in HBcrAg levels in the ETV continuation and ETV–TAF switch groups. The reduction in HBcrAg was significantly greater in the ETV-TAF group than in the ETV group at 2 and 3 years after switching. * *p* < 0.05, ETV, entecavir; TAF, tenofovir alafenamide; HBcrAg, hepatitis B core-related antigen. Boxes represent the interquartile range (IQR), with the center line indicating the median. Each diamond represents an individual patient.

HBV DNA levels remained suppressed at undetectable levels in both groups throughout follow-up, with no significant changes over time. In contrast, HBcrAg levels showed a significantly greater reduction in the ETV-TAF switch group than in the ETV continuation group. At year 2, the median change was −0.20 log U/mL (IQR −0.30 to −0.20) in the ETV–TAF switch group compared with −0.10 log U/mL (IQR −0.20 to 0.10) in the ETV continuation group (*p* = 0.007). At year 3, the corresponding median changes were −0.30 log U/mL (IQR −0.30 to −0.20) in the ETV–TAF switch group compared with −0.10 log U/mL (IQR −0.20 to 0.10) in the ETV continuation group (*p* = 0.006) (Figure 1, Table 3).

**Figure 2:**
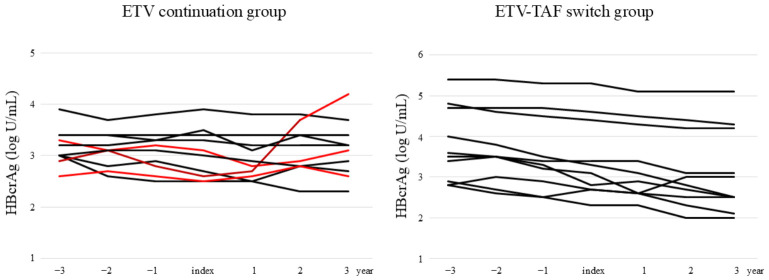
Individual trajectories of HBcrAg levels in 10 patients in the ETV continuation group and 10 patients in the ETV–TAF switch group. Black lines connect paired data from the same patient. In the ETV continuation group, three patients showed no decrease in HBcrAg levels at 3 years from the index year, whereas all patients in the ETV–TAF switch group exhibited a decline (indicated by red lines). ETV, entecavir; TAF, tenofovir alafenamide; HBcrAg, hepatitis B core-related antigen.

Analysis of individual HBcrAg trajectories showed that the HBcrAg levels did not decrease over the 3-year period from the index year in three of 10 patients in the ETV continuation group, whereas all patients in the ETV–TAF switch group exhibited a decline in HBcrAg levels after switching to TAF (Figure 2). Among the three patients in the ETV continuation group, one showed an adherence rate of <90%, whereas the remaining two maintained adherence rates of ≥90% based on a review of their medical records. No patients exhibited re-detectable HBV DNA during follow-up.

## 4. Discussion

In the current study, switching from long-term ETV therapy to TAF was associated with a greater reduction in HBcrAg levels in patients with chronic hepatitis B. Notably, further reductions in HBcrAg were observed even in patients with suppressed HBV DNA, suggesting an effect on viral transcriptional activity. Although the underlying mechanism remains unclear, these findings raise the possibility that TAF may influence not only viral replication but also cccDNA-derived transcription.

During HBV infection, cccDNA within hepatocyte nuclei serves as a template for viral transcripts, including pre-genomic RNA, and plays a central role in viral replication. Since cccDNA persists long-term in infected hepatocytes, it is a key factor in chronic infection and viral reactivation after treatment. Although serum HBV DNA and HBsAg levels are widely used for diagnosis and monitoring [16], they do not necessarily reflect cccDNA quantity or transcriptional activity during NA therapy, as circulating HBV DNA is rapidly suppressed while cccDNA persists. Consequently, these conventional markers have limited utility for assessing intrahepatic viral dynamics.

HBcrAg has recently emerged as a surrogate serum marker reflecting cccDNA transcriptional activity and has been shown to correlate with both cccDNA levels and activity, even during NA therapy [17,18,19]. Furthermore, HBcrAg remains detectable in patients with persistently undetectable HBV DNA and has been identified as an independent risk factor for HCC [9]. In this context, our study focused on HBcrAg as a marker of cccDNA activity and demonstrated differential longitudinal changes between continued ETV therapy and switching to TAF.

Previous studies have reported inconsistent findings regarding HBsAg reduction after switching from ETV to TAF, with some reporting comparable effects [20] and others suggesting greater reductions with TAF. Greater declines in HBsAg have been reported in non-cirrhotic patients and in those with lower baseline HBcrAg (<3.0 log U/mL) [21], as well as in patients with HBsAg levels < 800 IU/mL [22]. In addition, switching to TAF has not been associated with increased adverse events [23,24,25].

However, data on HBcrAg dynamics are limited. Itokawa et al. reported modest decreases in HBcrAg (approximately −0.1 log U/mL) at 48 weeks in both ETV continuation and switching groups, with no significant between-group difference [21]. The relatively short follow-up period of that study may have prevented detection of longer-term effects. Consistent with this, no significant difference was observed at 1 year in our study, whereas significant differences emerged at years 2 and 3, suggesting that longer follow-up is required to detect changes in HBcrAg.

Importantly, our study demonstrated differences in HBcrAg despite persistently undetectable HBV DNA levels, suggesting that HBcrAg captures differences in viral transcriptional activity not reflected by conventional viral load markers and supporting its utility as a complementary marker of viral control during NA therapy.

The current study has several important limitations. First, the single-center setting, retrospective design, and extremely small sample size limit the statistical power and generalizability of the findings. Second, although propensity score matching was performed to reduce baseline imbalance, multivariate analyses were not conducted because of the limited number of patients, and residual confounding may remain. Third, the treatment effects may have been influenced not only by pharmacological differences but also by adherence. In fact, one patient in the ETV group with adherence < 90% showed an increase in HBcrAg levels during follow-up. Because TAF can be administered without strict fasting requirements, it may facilitate better adherence, which could have contributed to the observed effects. Fourth, the inclusion of post-liver transplantation cases may have introduced heterogeneity. However, no patient received HBIG after the index year or after switching to TAF, suggesting a limited impact on study outcomes. In addition, all patients received tacrolimus, minimizing variability in immunosuppression. Notably, sustained reductions in HBcrAg were observed even in a cohort including a substantial proportion of liver transplant recipients, further supporting the potential benefit of TAF. In addition, external validation was not performed. Finally, whether reductions in HBcrAg translate into long-term reductions in hepatocarcinogenesis is unclear, warranting investigation in larger multicenter cohorts with longer follow-up periods. Therefore, these findings should be interpreted as exploratory and hypothesis-generating.

## 5. Conclusions

This study showed that switching from ETV to TAF over 3 years was associated with greater reductions in HBcrAg levels, including in post-liver transplant patients under immunosuppressive conditions. Although the clinical significance of HBcrAg reduction requires further clarification, switching to TAF may represent a clinically relevant strategy to suppress cccDNA transcriptional activity and achieve deeper viral suppression beyond HBV DNA negativity.

## Figures and Tables

**Table 1:** Baseline characteristics of patients in the ETV continuation and ETV–TAF switch groups.

Variable	ETV Continuation Group (*n* = 19)	ETV–TAF Switch Group (*n* = 15)	*p* Value
Age (years)	65 (57–69)	65 (58–72)	0.638
Sex (male/female)	14/5	15/0	0.053
HBsAg (IU/mL)	10.2 (0.05–851.7)	0.05 (0.05–11.6)	0.107
HBeAg (positive/negative)	4/15	3/12	1.000
HBcrAg (log U/mL)	3.3 (2.8–4.1)	2.9 (2.7–3.5)	0.207
HBV DNA (log IU/mL)	0	0	1.000
Platelet count (×10^4^/µL)	17.1 (11.6–18.7)	15.9 (13.6–19.8)	0.610
Prothrombin time (%)	92 (80–104)	92 (73–108)	0.980
Prothrombin time–INR	1.05 (0.98–1.10)	1.05 (0.97–1.45)	0.723
Albumin (g/dL)	4.4 (3.8–4.5)	3.9 (3.6–4.2)	0.109
eGFR (mL/min/1.73 m^2^)	67.9 (63.3–74.1)	62.2 (57.9–68.3)	0.092
Total bilirubin (mg/dL)	0.8 (0.7–1.1)	0.9 (0.7–1.1)	0.806
AST (U/L)	21 (19–30)	21 (13–30)	0.403
ALT (U/L)	20 (13–33)	21 (13–30)	0.794
FIB-4 index	2.15 (1.51–2.82)	1.74 (1.34–2.74)	0.455
Duration of ETV treatment until index year (years)	70 (50–88)	98 (63–110)	0.065
Post-liver transplantation, *n* (%)	5 (26)	10 (67)	0.018

Data are presented as the median (interquartile range) or number (%). ETV, entecavir; TAF, tenofovir alafenamide; HBsAg, hepatitis B surface antigen; HBeAg, hepatitis B e antigen; HBcrAg, hepatitis B core-related antigen; HBV DNA, hepatitis B virus DNA; eGFR, estimated glomerular filtration rate; AST, aspartate transaminase; ALT, alanine transaminase; FIB-4, fibrosis-4 index.

**Table 2:** Baseline characteristics of patients in the ETV continuation and ETV–TAF switch groups after propensity score matching.

Variable	ETV Continuation Group (*n* = 10)	ETV–TAF Switch Group (*n* = 10)	*p* Value
Age (years)	68 (64–70)	65 (57–72)	0.753
Sex (male/female)	10/0	10/0	1.000
HBsAg (IU/mL)	6.13 (0.05–164)	4.30 (0.05–975)	0.842
HBeAg (positive/negative)	0/10	3/7	0.210
HBcrAg (log U/mL)	3.2 (2.8–3.4)	3.0 (2.7–4.4)	0.985
HBV DNA (log IU/mL)	0	0	1.000
Platelet count (×10^4^/µL)	15.3 (10.4–17.8)	15.9 (13.3–20.6)	0.393
Prothrombin time (%)	91 (84–103)	92 (76–109)	0.906
Prothrombin time–INR	1.06 (0.99–1.09)	1.05 (0.98–1.39)	0.979
Albumin (g/dL)	4.2 (3.6–4.5)	4.2 (3.8–4.5)	0.985
eGFR (mL/min/1.73 m^2^)	66.5 (59.9–68.0)	65.6 (61.2–71.1)	0.796
Total bilirubin (mg/dL)	1.0 (0.7–1.2)	0.7 (0.7–0.9)	0.197
AST (U/L)	21 (19–30)	20 (16–23)	0.393
ALT (U/L)	20 (18–34)	17 (13–31)	0.247
FIB-4 index	2.45 (1.50–3.24)	1.66 (1.30–2.57)	0.165
Duration of ETV treatment until index year (years)	75 (60–107)	82 (45–112)	0.911
Post-liver transplantation, *n* (%)	4 (40)	5 (50)	1.000

Data are presented as median (interquartile range) or number (%). ETV, entecavir; TAF, tenofovir alafenamide; HBsAg, hepatitis B surface antigen; HBeAg, hepatitis B e antigen; HBcrAg, hepatitis B core-related antigen; HBV DNA, hepatitis B virus DNA; eGFR, estimated glomerular filtration rate; AST, aspartate transaminase; ALT, alanine transaminase; FIB-4, fibrosis-4 index.

**Table 3:** Longitudinal changes in HBcrAg (log U/mL) from the index year.

Time Point	EVT Continuation Group	ETV-TAF Switch Group	*p* Value
Year 1	−0.10 (−0.20 to 0)	−0.10 (−0.20 to 0)	0.796
Year 2	−0.10 (−0.20 to 0.10)	−0.20 (−0.30 to −0.20)	0.007
Year 3	−0.10 (−0.20 to 0.10)	−0.30 (−0.30 to −0.20)	0.006

Data are presented as median (interquartile range). Values indicate changes in HBcrAg (log U/mL) from the index year. Negative values indicate a reduction. *p* values were calculated using the Mann–Whitney U test. ETV, entecavir; TAF, tenofovir alafenamide; HBcrAg, hepatitis B core-related antigen.

## Data Availability

The data presented in this study are available upon request from the corresponding author. These data are not publicly available because they contain sensitive medical information.

## Abbreviations

The following abbreviations are used in this manuscript:

cccDNA	covalently closed circular DNA
ETV	Entecavir
HBcrAg	Hepatitis B core-related antigen
HBIG	hepatitis B immunoglobulin
HBsAg	hepatitis B surface antigen
HBV	Hepatitis B virus
HCC	hepatocellular carcinoma
NAs	nucleos(t)ide analogs
TAF	tenofovir alafenamide
TDF	tenofovir disoproxil fumarate
